# Experimental and numerical analysis on cold forging of commercially pure aluminum

**DOI:** 10.1038/s41598-026-37220-8

**Published:** 2026-02-02

**Authors:** Khemraj Sahu, Manvandra Kumar Singh, Hemant Kumar Choudhary, Raj Bahadur Singh, Jitendra Kumar Katiyar

**Affiliations:** 1 Department of Automation and Robotics, SIT College of Engineering, Yadrav (Ichalkaranji) Kolhapur, Maharashtra 416121 India; 2 Department of Mechanical Engineering, Graphic Era (Deemed to be University), Dehradun, Uttarakhand 248002 India; 3 Department of Mechanical Engineering, Muzaffarpur Institute of Technology, Muzaffarpur, Bihar 842003 India; 4 Department of Materials Science and Engineering, National Institute of Technology, Hamirpur, Himachal Pradesh 177005 India; 5 Manipal Institute of Technology, Manipal Academy of Higher Education, Manipal, Karnataka 576104 India

**Keywords:** Near net shape, Forging, Commercially pure aluminum, Finite element analysis, Friction, Engineering, Materials science, Mathematics and computing

## Abstract

Near net shape forging represents a manufacturing philosophy aimed at achieving components that closely approximate their final geometries in the as-forged state. Unlike a specific forging process, it emphasizes minimizing post-forging machining and material waste while enhancing efficiency and cost-effectiveness. The present work focuses on the preform design of commercially pure aluminium for closed-die forging conditions by analyzing its flow in the die cavity using Deform 3D software. The simulations have been conducted using DEFORM 3D V11.2 software to determine the optimal shape and size of the preform, thereby minimizing costly and inefficient shop floor iterations. The fully defined finite element model includes tetrahedral elements, refined contact region mesh, Lagrangian incremental solver, Coulomb friction formulation, and a calibrated nonlinear hardening law. Three preform geometries of equal volume were evaluated to determine the most suitable design for forming a 40 mm sphere. Experiments validated numerical predictions using a closed-die forging setup. Results show strong agreement in material flow, die filling, and energy trends, though quantitative deviations in forging energy (12.8%), ovality (1.9% simulation vs. 3.6% experimental), die-filling completeness (97.4% simulation vs. 95.1% experiment), and volume conservation (< 1% error in both cases) arose due to friction and strain hardening effects. The findings highlight the effectiveness of simulation in refining the forging process, offering practical insights for manufacturing complex components with improved quality and efficiency.

## Introduction

The term near net shape forging refers to a conceptual approach rather than a specific forging process. Its goal is to produce a component that closely resembles the final shape in its as-forged condition. Net shape indicates no additional machining or finishing is needed on the forged surfaces, though secondary operations may be required for minor features like holes, threads, or similar details^1,2^.

In the current scenario, the near net forging is very popular in different industries such as automotive, aerospace, marine and structural parts of ferrous and non-ferrous materials. Among these, aluminum and its alloys have gained significant attention due to their ability to produce components with minimal post-processing, reducing material waste and improving efficiency. Aluminum alloys, such as AA6061, AA7075, and AA5083, are widely used in aerospace, automotive, and marine industries for their lightweight properties, excellent strength-to-weight ratio, and corrosion resistance^3–5^. Various studies were conducted on the mechanical properties of aluminum alloys produced via near-net shape forging, revealing improvements in strength and durability compared to traditional methods^6,7^. The study of near-net shape forging of aluminum alloys focuses on the impact of processing parameters on material properties and the potential for reducing manufacturing waste^8^. Hot forging of high-strength aluminum alloys highlights the ability to achieve near-net shapes with enhanced mechanical performance^9,10^.

Recent developments in metal forming technologies use various FEM-based simulation tools to optimize process parameters to confirm the exact dimensions of the product and improve mechanical characteristics. The FEM simulation tools offered the ability to analyze the metal flow, temperature distribution, and defect formation during the process. The outcomes of the simulation analysis allowed manufacturers to plan systematic processes and overcome the shortcomings.

Chen et al.^8^ used DEFORM 3D finite element simulation to look into how to design and make the I, L, and T-shaped molds for a 2A14 aluminum alloy part with intriguing shapes. The result reveals that L-shaped preforms showed optimum filling for the cavity, while other I- and T-shaped preforms suffered from folding defects. The experimental results also confirmed that an L-shaped preform, free from any forging defect, could fabricate a complex-shaped forged product. Feng et al.^11^ looked at how the shape of the die affected the deformation of a thin-walled 6A02 aluminum alloy during an isothermal forging process. The study found that the optimal die profile ensured a filled die cavity with minimal die flash and higher dimensional accuracy of the product. The experimental results indicated that a higher forging load was needed to deform the material because the test samples and the die wall surfaces were rubbing against each other.

Krishna and Jena^12^ showed an analytical and FEA-based way to figure out how much force is needed to forge AA6063, mild steel, and brass billets with circular and elliptical cross-sections. Results show the forging force is higher for circular billets and increases with billet volume. FEA simulations, accurate within 5% error, confirm the analytical model’s validity for industrial applications, though limitations exist for height reduction below 0.3. Konstantinov et al.^13^ performed a numerical simulation to explore the various processing parameters for hot forging 5083 aluminum alloy. In this experiment, they optimized the forging temperature, strain rate, and deformation power during isothermal forging. They reported that through computer simulation using DEFORM-3D software, a novel combined sheet stamping and hot die forging process was developed, allowing two forgings to be produced per press stroke. The study confirmed that the new process could be implemented without the need for equipment replacement, with only minor adjustments such as incorporating an isothermal block and modifying the stamping insert.

The main findings from the above studies highlight the challenges of achieving near-net forging in commercially pure aluminum, especially in closed-die forging. The present work focuses on designing preforms to produce near-net shape products. A comparative evaluation of three preform geometries of identical volume was conducted. The spherical segment radii of 30 mm, 35 mm, and 40 mm were compared based on energy consumption and die filling behaviour. The performance volume and material flow in the die cavity were analyzed using DEFORM-3D V11.2, and the simulation results were validated experimentally.

## Numerical simulation scheme and methodology

**Fig. 1:**
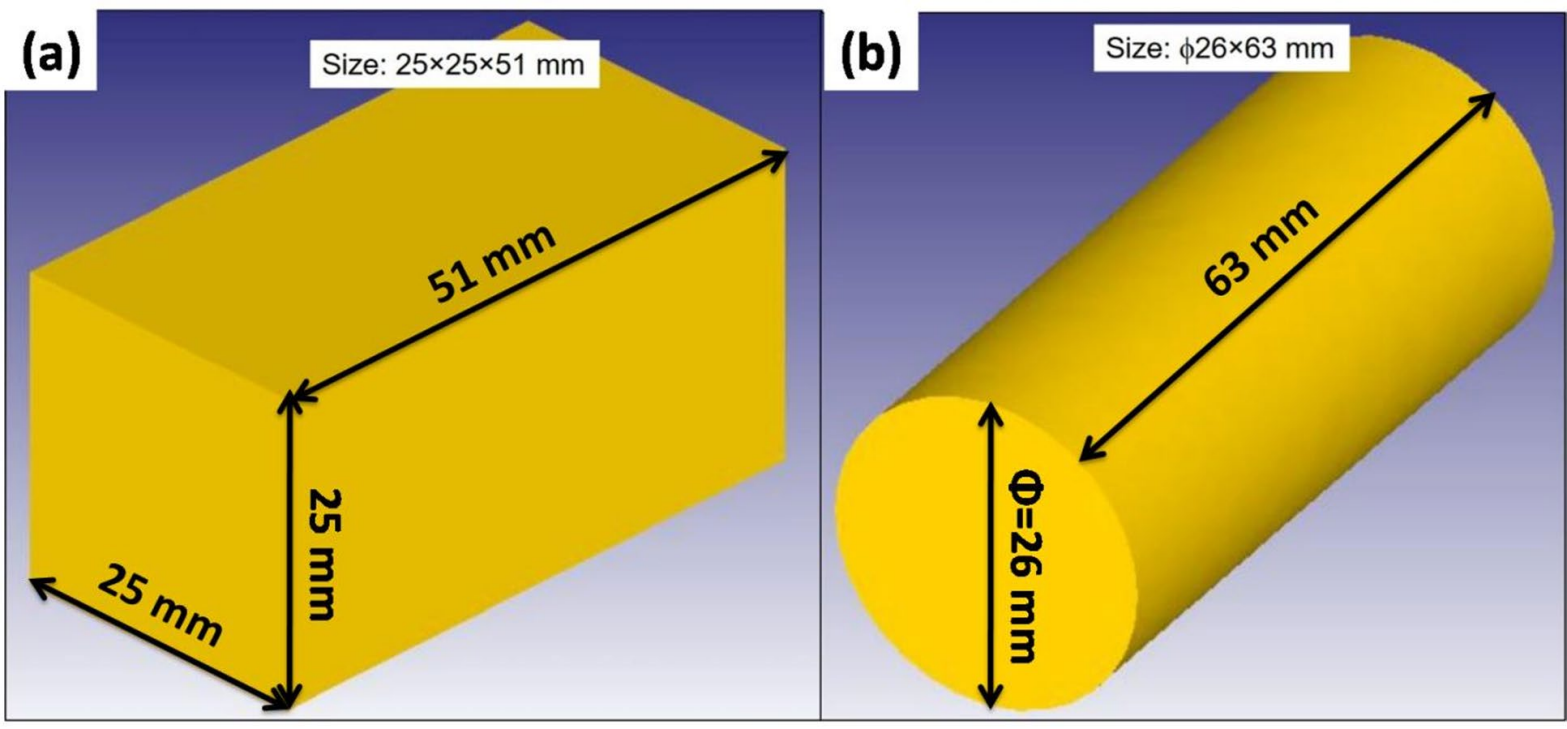
Dimensions of the preform (**a**) Rectangular shape, (**b**) Cylindrical shape.

**Fig. 2:**
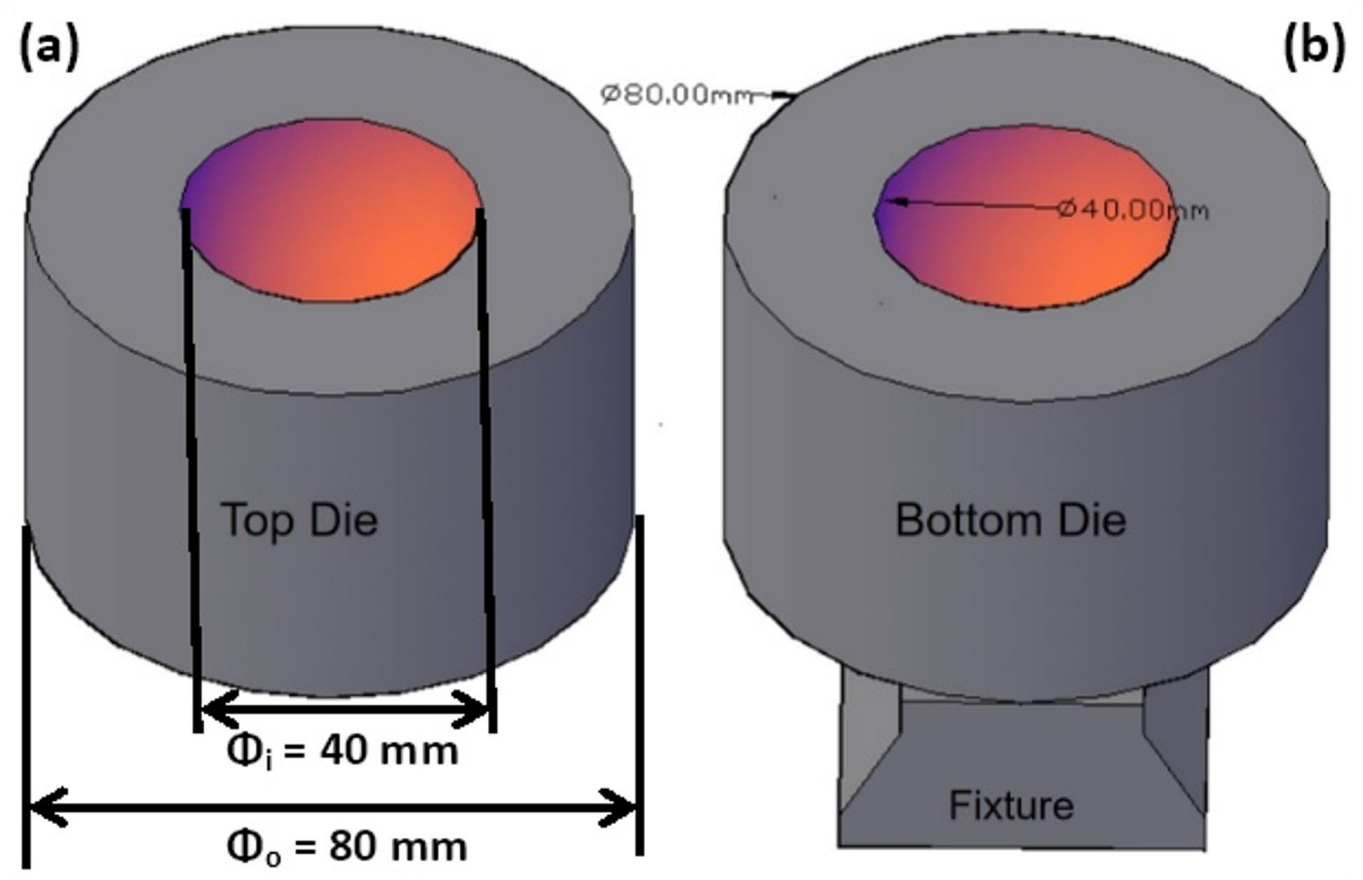
Closed-die setup with fixture arrangement.

The cold forging process was modelled both axis-symmetrically and plane-symmetrically using the commercial finite element software Deform 3D. The preform was defined as an axisymmetric deformable solid cylinder (Fig. 1a) and a plane-symmetric (Fig. 1b) deformable solid rectangle, while the die was represented as a hollow spherical shape having a diameter of 40 mm, as shown in Fig. 2.

**Fig. 3:**
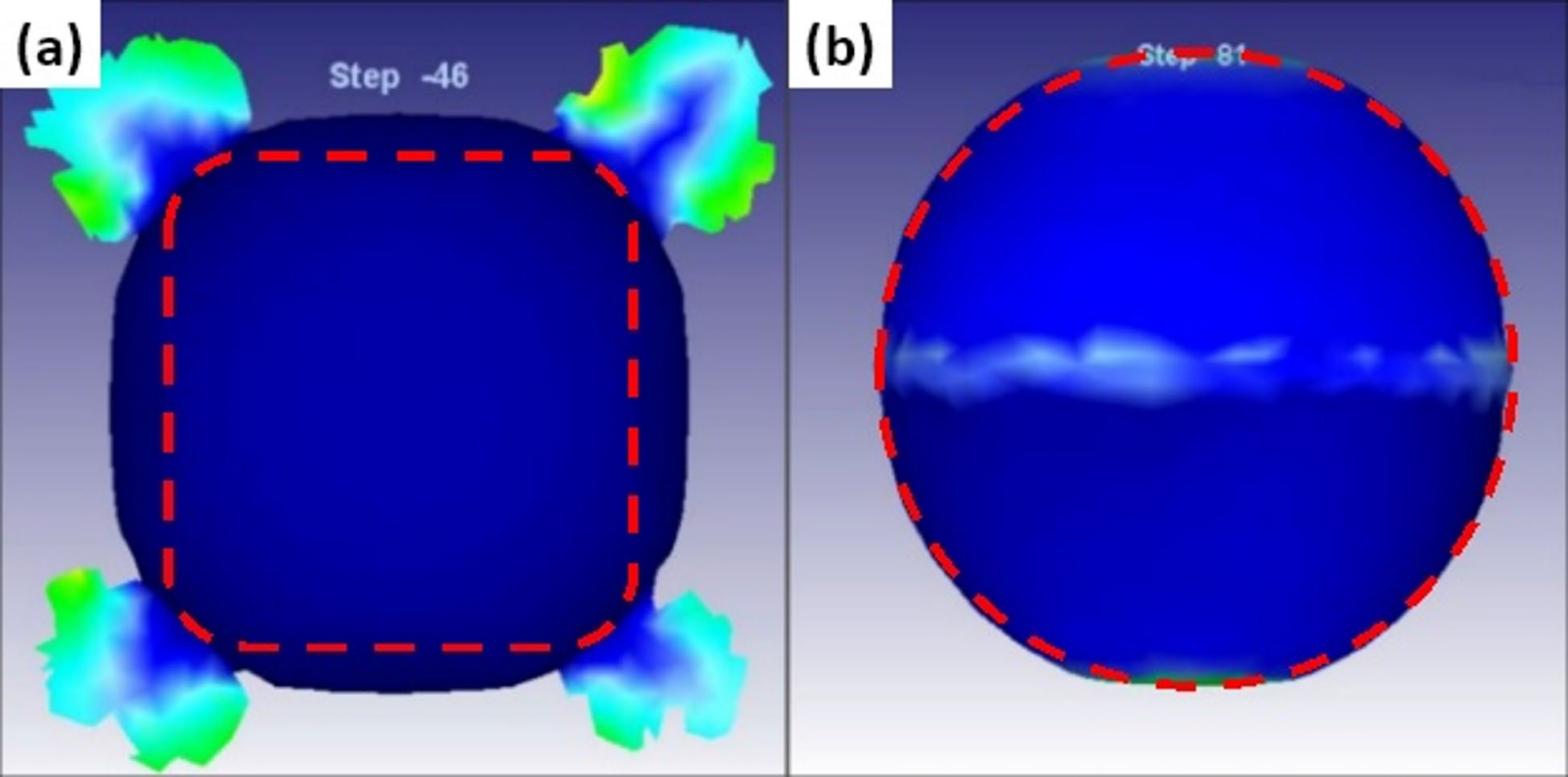
Simulation of material flow in the die cavity (**a**) Rectangle shape, (**b**) Cylindrical shape.

The dimensions of the preform were systematically derived by conducting a series of simulations of the forging process based on the minimum energy required to fill the die cavity without a flash. Figure 3 displays the cold forging simulation results for both rectangular and cylindrical preforms. The results reveal that when a rectangle-shaped preform deforms in the die cavity, excessive flash occurs at the corners, as shown in Fig. 3(a), and the final shape is not a sphere. This condition renders the rectangle-shaped preform unsuitable for generating a sphere-shaped product, leading to its discard. Figure 3(b) shows the simulation result for forging a cylindrical-shaped preform, and it indicates that minor flash occurs at the die junction. The final shape approaches a sphere except for flat poles. During the forging process, the velocity vectors of the metal flow are directed outwards at the poles, even when the ends are not properly filled. This sign indicates that the end part of the die cavity was not filled, and there is a vacant space. If we give the preform metal in excess, the flash will occur at the die junction. Therefore, the form design ensures the transfer of the flash material to the poles. A convenient option for this purpose is to provide a suitable end radius. The value of the end radius is determined by simulating preforms having different end radii and comparing them for the minimum energy criterion.

The dimensions of the preform were chosen to match the volume of the die cavity, except for varying the spherical segment in the preform. The preform was divided into three sections in which the diameter of the cylinder part was 26 mm, the height was 59 mm, and the radius of the spherical segment was 40, 35, and 30 mm. Therefore, die was modeled as a rigid body containing a 40 mm spherical cavity. Three preform geometries with identical volume but spherical radii of 30, 35, and 40 mm were evaluated. Before the simulation of the forging process, the finite element mesh is generated in the workpiece model, and the material properties are added to the library. The workpiece was discretized using tetrahedral Lagrangian elements with an average mesh size of 1.5 mm and 0.8 mm refinement along the die and workpiece interface. An Lagrangian incremental solver in DEFORM 3D was used for quasi-static cold forging. A Coulomb friction law with µ = 0.02 was applied, selected from literature for cold forging of pure aluminum^14^. The statistics of the mesh are summarized in Table 1 for spherical samples of various radii. As the radius increases from 30 mm to 40 mm, both element and node counts gradually rise, reflecting the higher mesh density required for larger geometries.

**Table 1:** Mesh statistics for sample different spherical radii.

S. no.	Samples with spherical radii (in mm)	Element	Node
1	30	51,464	80,246
2	35	53,300	83,035
3	40	55,706	85,143

**Fig. 4:**
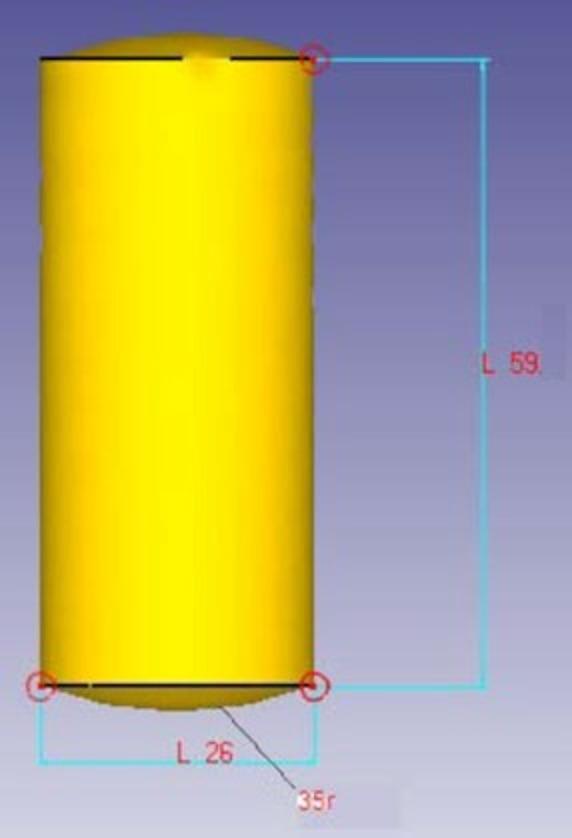
Model of the preform.

**Fig. 5:**
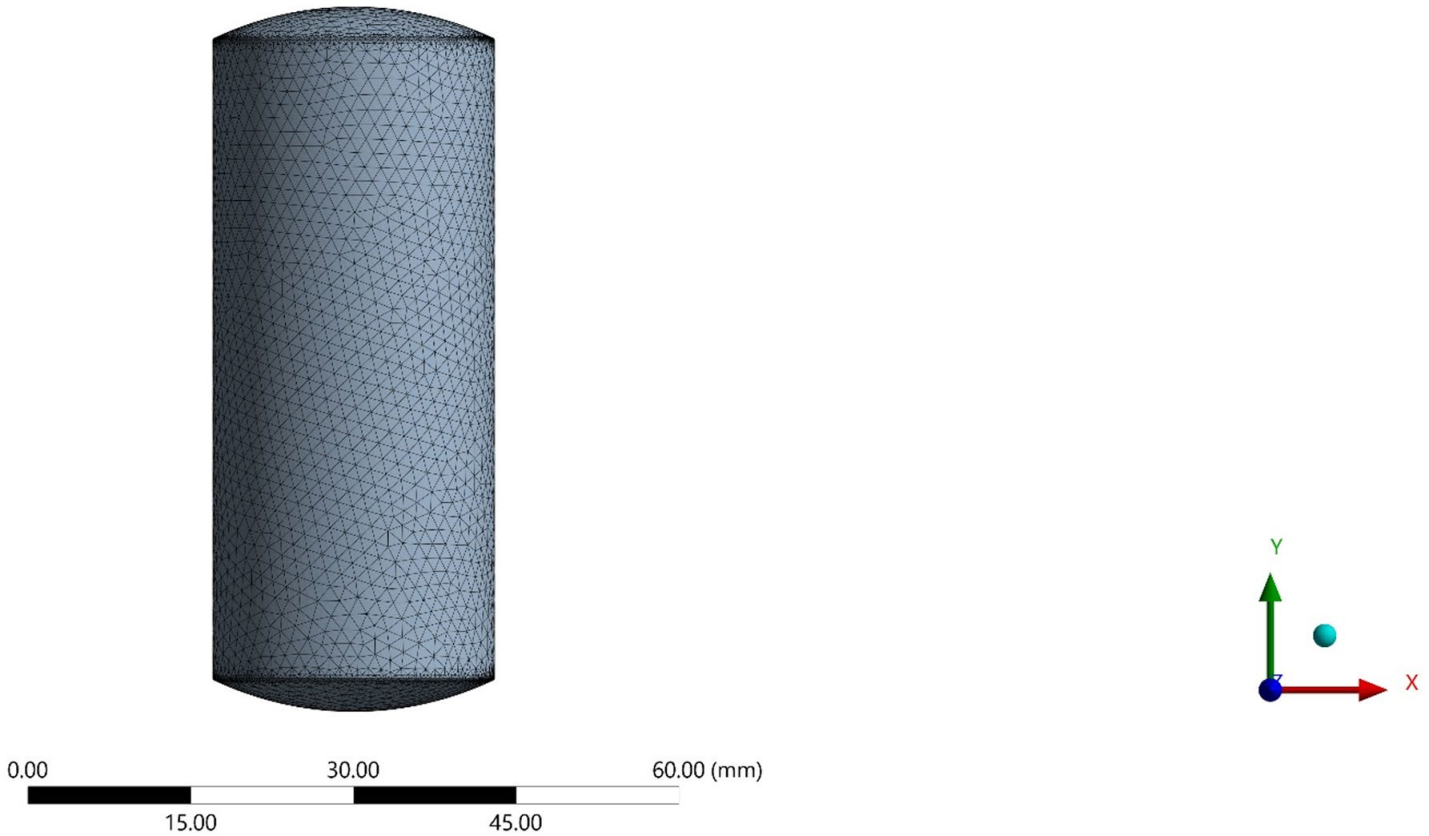
Meshed geometry of the spherical ended workpiece (*r* = 35 mm).

The simulation was performed at a strain rate of 1 mm/sec, and the top die moved downward while fixing the bottom die. A reference point was established on the die, through which all its motions and constraints were applied. Table 2 shows the mechanical properties of commercially pure 99.8% aluminum as the material for the preform. Commercially pure aluminum (99.8%) tensile data were obtained from manufacturer datasheets and verified by in house tensile tests. Plastic deformation was modelled using a Hollomon hardening law σ = Kεⁿ with K = 148 MPa and *n* = 0.21 derived via curve‑fitting. This nonlinear model was essential for accurately predicting strain‑dependent flow stress and forming load. Figure 4 shows the 3D model of the test preform. Figure 5 displays the generated meshed spherical-ended cylindrical workpiece employing tetrahedral Lagrangian elements, which presents a consistent mesh throughout its surface.

Table 2 shows the various mechanical properties of commercially pure aluminum (99.8%) for experimentation in this research work.

**Table 2:** Mechanical properties of the commercially pure aluminum (99.8%).

S. no.	Property	Values
1	Yield strength	34 MPa
2	Tensile strength	75 MPa
3	Density	2.7 g/cm³
4	Young Modulus	69 GPa
5	Elongation to break	34%

The material flow behavior during the initial and progressive stages of the cold forging process is a key aspect in determining the quality and completeness of die filling. At the beginning of the compression process, the metal experiences a downward force as the top die moves. This initial load causes the metal to flow in response to the applied pressure, primarily in a downwards and outwards direction. As observed from Fig. 5, the metal begins to spread radially outward along the surface, conforming to the shape of the die cavity. This radial outward flow is a characteristic feature of forging processes, where the material, being incompressible, must move to occupy available space when compressed axially. The radial flow not only facilitates the filling of the die cavity but also ensures uniform deformation if the die and preform are properly aligned. At this stage, the flow stress acting against the die wall is mild, indicating that the metal still has space to flow and is not yet fully constrained by the cavity.

**Fig. 6:**
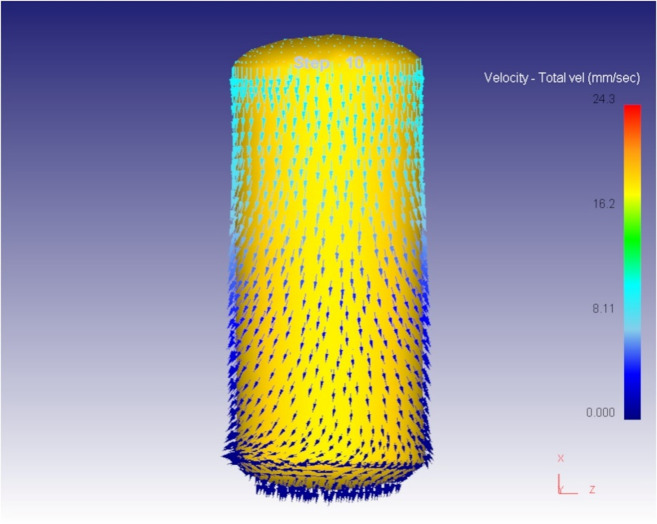
Metal flow in initial steps.

**Fig. 7:**
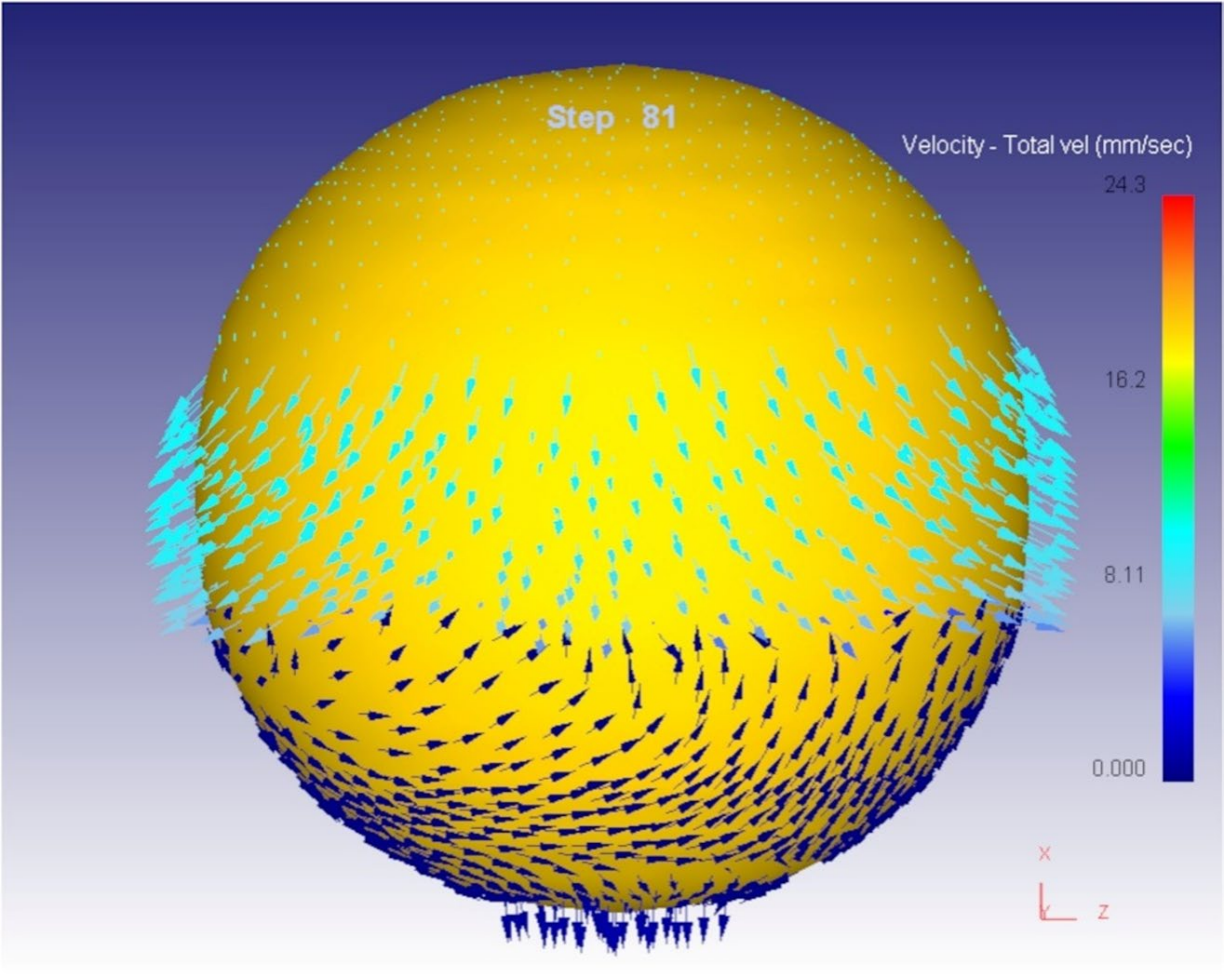
Final Step of the compression process.

As the compression continues, the available space within the die cavity decreases, and the metal starts to encounter greater resistance. Figures 6 and 7 illustrate the continuation and intensification of radial flow as the material spreads to fill the cavity. This outward flow is crucial for achieving a defect-free final shape, particularly in spherical or complex geometries. A critical observation is made at step 81 of the simulation, as shown in Fig. 6. At this point, the die cavity is filled with the deforming material. The development of back stress internal resistance generated in the material opposes the ongoing flow stress. This phenomenon marks the completion of the cavity filling process. The presence of back stress is a clear indication that the material has no additional space to flow into, and any further deformation would result in either densification, flash formation, or potential die overload.

In summary, the metal flow behavior observed during forging begins with smooth outward movement from axial compression, gradually leading to full cavity filling. The sequential development of stress, including opposing back stress, confirms the transition from free flow to complete filling. These insights are essential for advancing die design and preform geometry, ensuring that the final product meets dimensional accuracy and structural integrity requirements.

Figure 8(a-c) illustrates the relationship between deformation time and the energy required to fill the die cavity for three different preform configurations, each with varying spherical segment radii: 40 mm, 35 mm, and 30 mm. The graphs clearly show that, in all cases, the energy consumption increases linearly with time as deformation progresses. This linearity reflects a consistent resistance during the forming process, primarily due to the frictional effects and material work hardening experienced under cold forging conditions. Among the three spherical segment configurations, the preform with a 40 mm radius required the most energy to fill the die cavity completely. This can be attributed to the larger volume and more extensive contact area between the preform and die wall, which results in higher frictional resistance and more material displacement. In contrast, the energy required reduced progressively with smaller spherical segment radii. The preform with a 35 mm radius exhibited a moderate energy requirement, while the 30 mm radius configuration demanded the least energy.

This trend highlights a critical insight reducing the spherical segment radius leads to lower energy consumption during the forging process. A smaller spherical segment reduces the initial material volume to be deformed into the die cavity and minimizes surface contact with the die, thereby decreasing the frictional losses and internal resistance during the deformation. As such, forming efficiency improves with reduced spherical segment radii. The goal of this study was to comparative evaluation of the preform design based on the minimum energy consumption criterion, ensuring the forging process is both energy-efficient and capable of producing high-quality parts. Material flow analysis indicates outward radial spreading followed by die constraint-induced back stress. The 30 mm-radius preform produced smooth cavity filling and lowest forming load. Energy increased linearly with time due to friction and work hardening effects. All three preforms were simulated under identical boundary conditions. Among these tested preform, the one with a spherical segment radius of 30 mm proved to be the most effective. It not only consumed the least energy during the forging process but also resulted in a defect-free product with a nearly perfect spherical shape. Importantly, no flash formation was observed, which is essential for reducing materials waste and avoiding additional post-processing.

Consequently, the dimensions of the preform selected for further experimental validation were finalized based on this simulation results. The chosen dimensions were a cylindrical diameter of 26 mm, a total height of 59 mm, and a spherical segment radius of 30 mm. This combination met all the necessary conditions for an efficient and controlled cold forging operation, minimizing energy input while achieving a well-formed product without surface defects or dimensional inaccuracies. Overall, the energy time relationship and corresponding outcomes validate that careful design of preform geometry, especially the spherical segment radius, plays a crucial role in enhancing the efficiency and effectiveness of the cold forging process. This approach ensures not only energy savings but also superior product quality, making it highly relevant for industrial applications where cost-efficiency and precision are critical.

**Fig. 8a:**
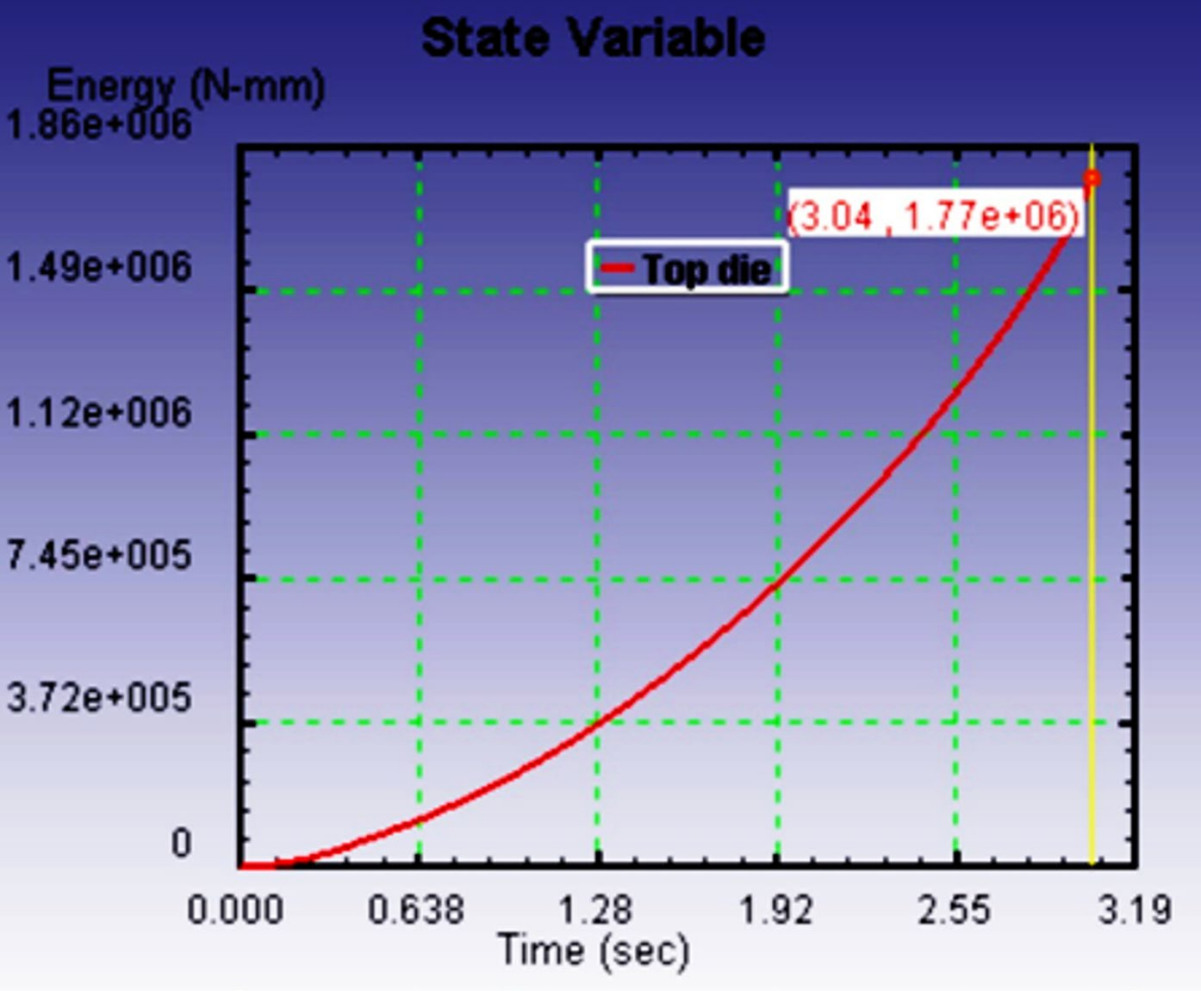
Energy required to fill the die cavity with respect to time for *r* = 40 mm.

**Fig. 8b:**
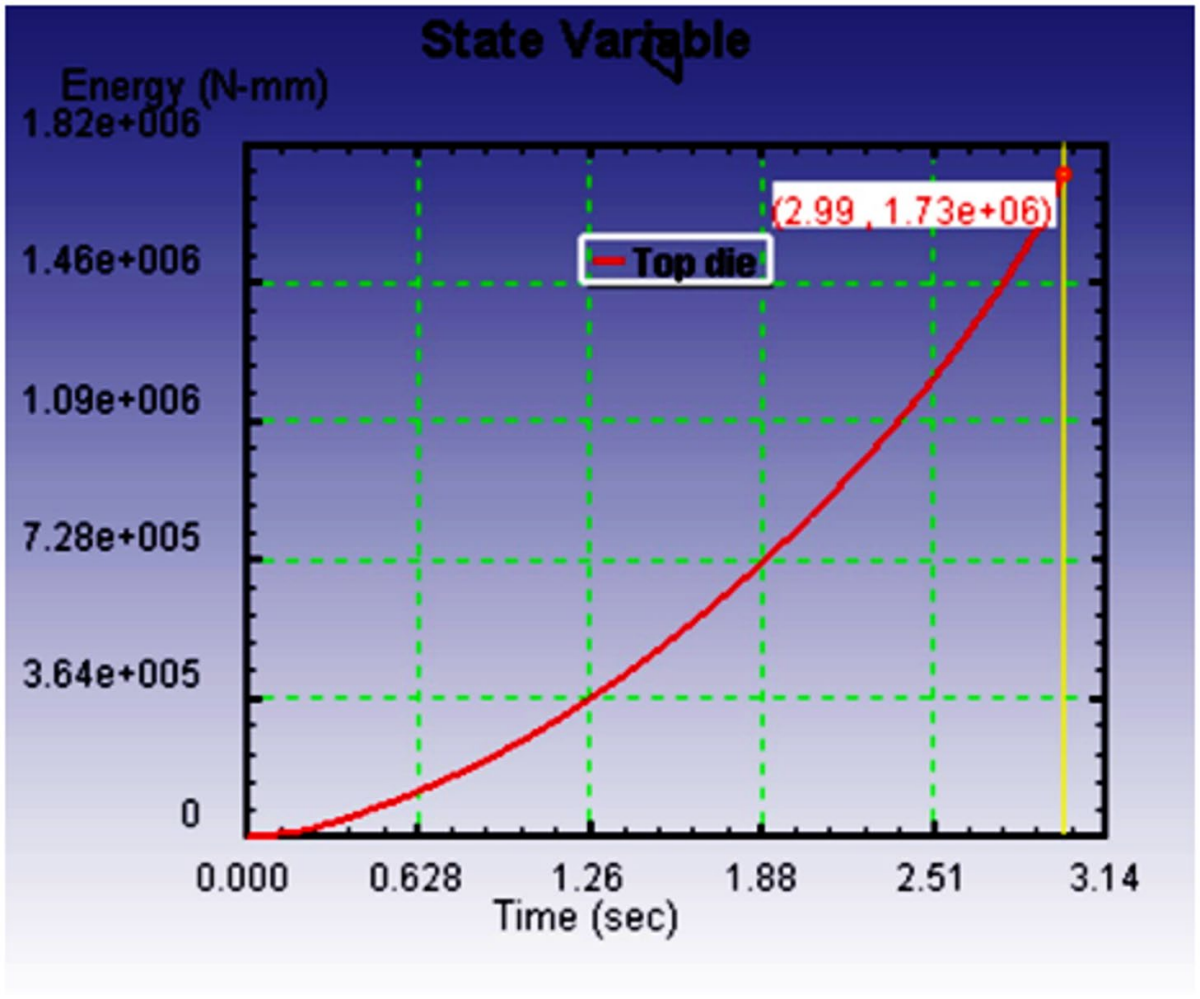
Energy required to fill the die cavity with respect to time for *r* = 35 mm.

**Fig. 8c:**
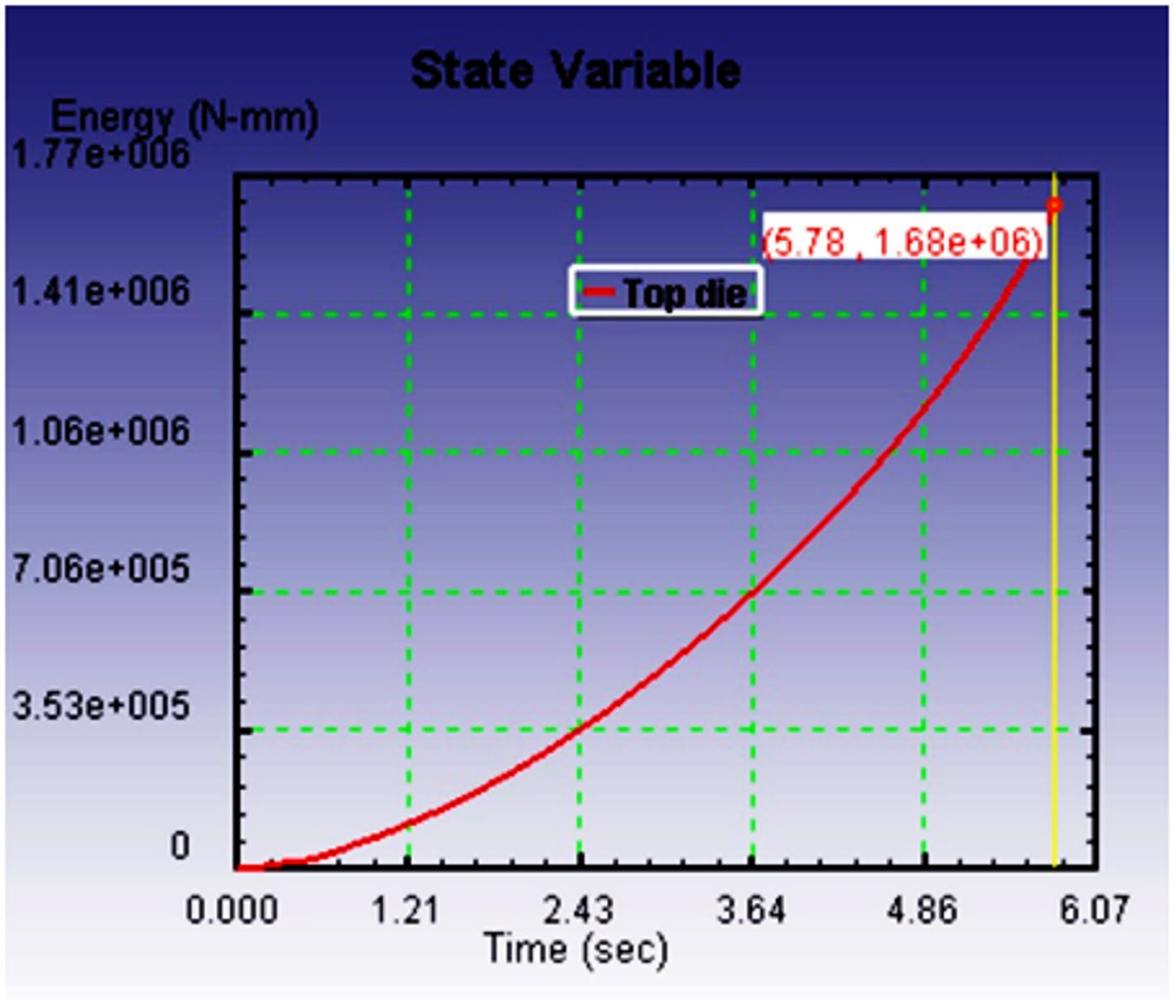
Energy required to fill the die cavity with respect to time for *r* = 30 mm.

## Experimental validation

To confirm the accuracy and reliability of the simulation results, a physical forging experiment was carried out under closed die conditions at room temperature, replicating the cold forging environment. This experimental validation is a crucial step in finite element modelling, as it helps ensure that the theoretical and simulated predictions align with actual physical behavior under similar conditions. The experimental setup involved the use of a closed die forging system. The dies were fabricated from H13 die steel, a material known for its high toughness, excellent heat resistance, and durability, making it ideal for withstanding the pressures involved in forging operations. In order to maintain accurate die alignment and prevent lateral movement or misalignment during the forging stroke, a guiding arrangement made from medium carbon steel was incorporated. This guidance system played a significant role in ensuring the precision and uniformity of the forging process.

The test samples used in the experiment were prepared from commercially pure aluminum billets. Aluminum was chosen due to its excellent formability and relevance to the simulation. The billets were machined to meet specific dimensional requirements: a cylindrical base with a height of 59 mm and a diameter of 26 mm, and a spherical segment of 30 mm radius. These dimensions matched the preform geometry used in the simulation, ensuring consistency in comparison between simulation and experiment. The actual forging was carried out using a 100 ton capacity universal testing machine, capable of providing sufficient force to deform the aluminum sample under cold working conditions. Before the experiment, all components, including the die cavities and aluminum samples, were thoroughly cleaned with acetone. This step was essential to remove any impurities, oil, or residues that might influence the forging process or compromise the quality of the finished product.

**Fig. 9:**
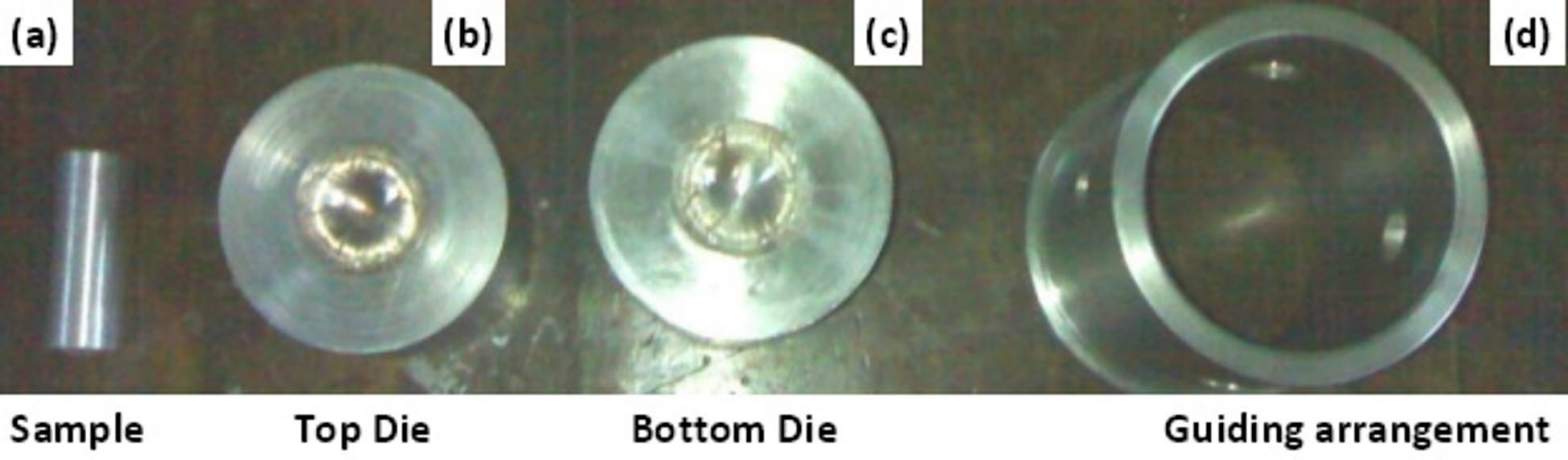
Photograph of the die setup and billet.

Figure 9 captures a photograph of the complete setup, showcasing the die assemblies and test sample before the initiation of the forging operation. Following the forging, the forged parts were examined to assess material flow behavior and the overall forming conditions. Observations included evaluating whether the die cavity was filled properly, the extent of flash generation, and the presence of any defects. These experimental findings were used to validate and refine the simulation outcomes, reinforcing confidence in the modelled forging process and its ability to predict real-world performance.

**Fig. 10:**
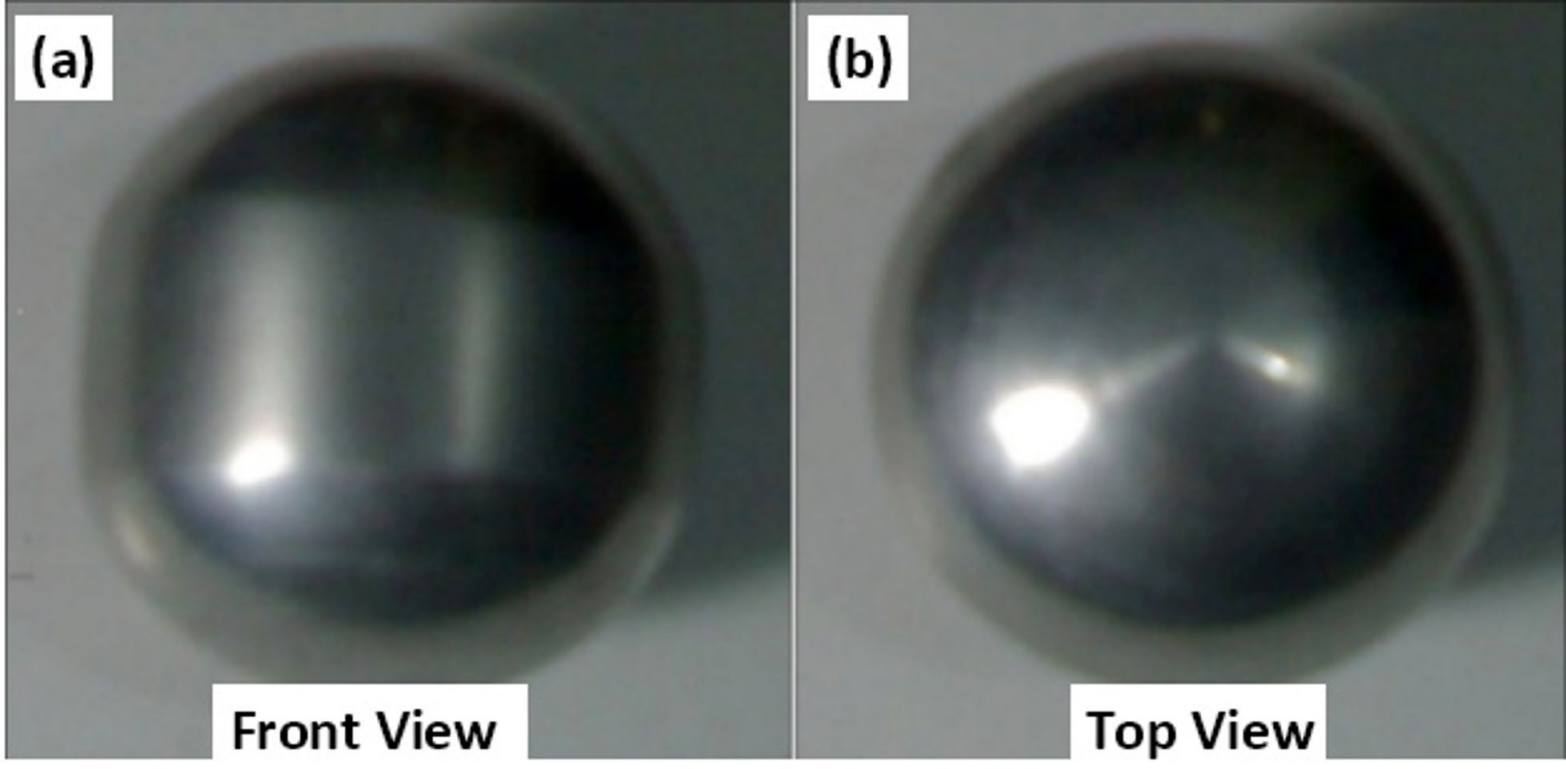
Photographs of the forged product.

Figure 10 presents both the front and top views of the forged product resulting from the cold forging simulation. Upon examining the visual output, it becomes evident that the final product does not achieve a perfectly spherical geometry; instead, it displays noticeable ovality. This deviation from the ideal shape is attributed primarily to the frictional interactions between the die wall and the deforming workpiece during the forging process^15–18^. In cold forging, friction plays a crucial role in shaping the final product, and in this case, it has restricted the uniform material flow required to form a perfect sphere. When the metal is forced into the die cavity, ideally, it should distribute evenly in all directions to achieve spherical symmetry. However, due to the resistance offered by the die walls through friction, the flow of materials is hindered in certain directions, especially near the interface where contact pressure is high. This frictional constraint causes the material to flow non-uniformly, leading to shape distortion and, in this case, an oval-shaped cross-section rather than a round sphere.

Moreover, the problem is further intensified by the nature of the cold forging process. In cold working, the material is deformed at room temperature, or slightly above, which enhances strain hardening. As the workpiece undergoes plastic deformation, its internal resistance to further deformation increases. This strain hardening contributes to increased friction at the die-workpiece interface because the harder the material becomes, the more resistant it is to sliding against the die surface. Therefore, as the process continues, the friction coefficient rises, compounding the non-uniformity in material flow.

Another aspect worth considering is the potential temperature influence. Although cold forging is performed at low temperatures to preserve dimensional accuracy and surface quality, it comes with the trade-off of reduced ductility and increased flow resistance. Unlike hot forging, where elevated temperatures promote metal flow and grain refinement, cold forging limits the material’s mobility, further amplifying the effect of friction. This condition results in incomplete or uneven filling of the die cavity, contributing again to shape irregularity such as ovality. Despite the geometric imperfection, the forged product exhibits a satisfactory surface finish. This is a typical advantage of cold forging, where the absence of oxidation and thermal effects during deformation often results in a smooth, defect-free surface. There are no visible cracks, laps, or surface tears, indicating that the forming process was effectively controlled and that the workpiece material possessed adequate ductility under the applied conditions. The surface quality is essential for components intended for mechanical applications, as surface integrity can directly affect fatigue strength and wear resistance.

**Fig. 11:**
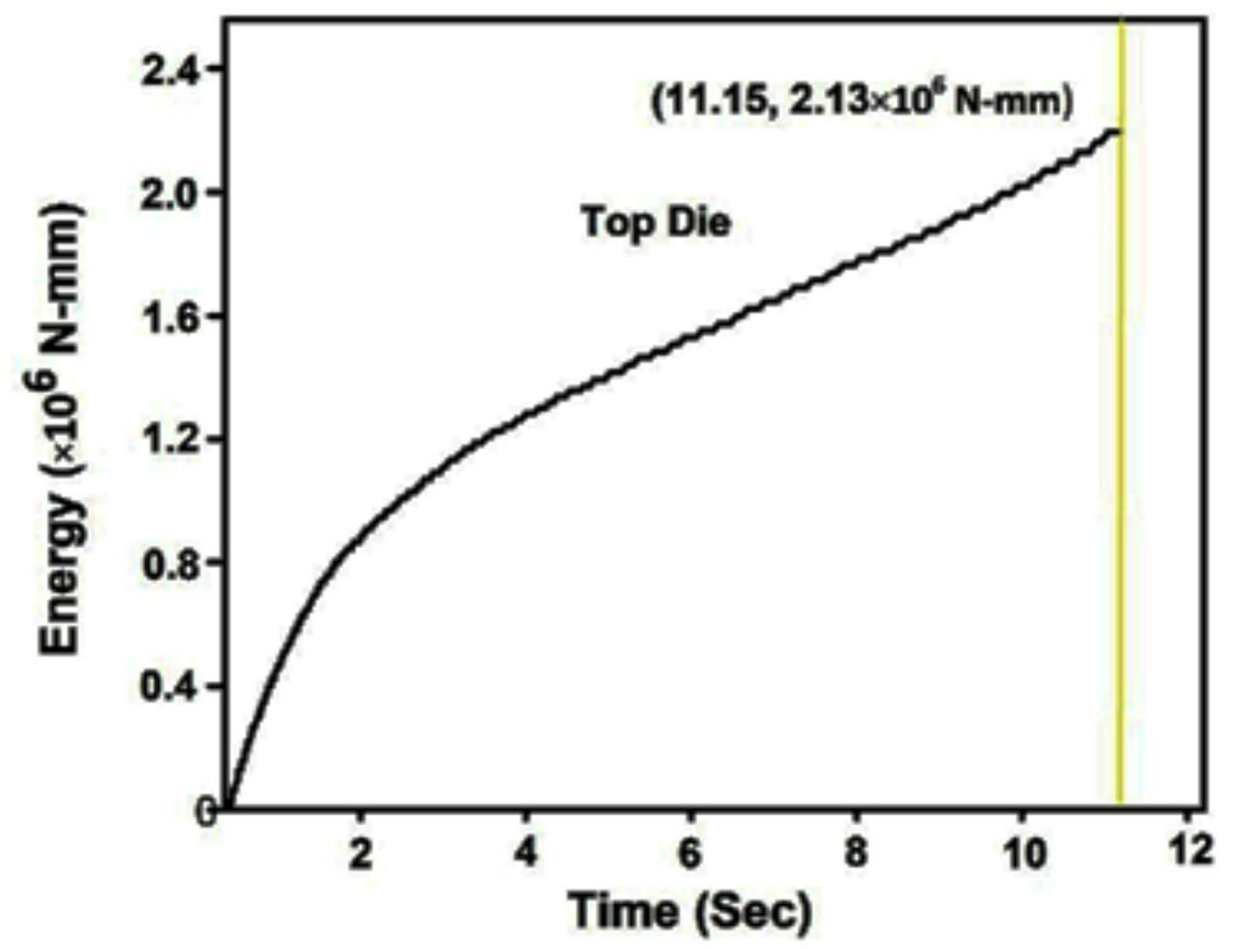
Relationship between the energy required against forging time.

Figure 11 shows the energy required against forging time to fill the die cavity. The result indicates that in actual forging conditions, the energy needed to fill the die cavity is initially more, and thereafter, it increases gradually. When comparing the actual forging result with the simulation result in Fig. 8c, the energy and time required to complete the forging process are higher. It may be due to strain hardening developed during the process, which opposes the forging load to deform the material and leads to more energy required to fill the die cavity. It may be due to strain hardening developed during the process, which opposes the forging load to deform the material and leads to more energy required to fill the die cavity. Essentially, the harder material resists the applied force more, requiring additional energy to overcome this increased resistance. The effect of strain hardening becomes more pronounced as deformation progresses, contributing to higher forces and energy consumption, which in turn makes it more difficult to fill the die cavity.

Another possible reason may be the friction generated between the preform and die wall, which restricts the flow of materials in the die cavity and offers more energy to proceed with the deformation.

Dixit et al.^15^ and Pop et al.^16^ have both previously reported similar findings, suggesting that friction plays a significant role in determining the energy required to deform a material. Specifically, they observed that as friction increases, the load or force required to deform the material also increases. These findings can be understood in the context of material deformation, where friction between surfaces or within the material itself contributes to additional resistance during the deformation process. The increased friction leads to greater energy dissipation, making it more challenging to deform the material and, consequently, raising the deformation load. Essentially, when friction is high, more energy is needed to overcome the resistance it creates, thereby increasing the force required for deformation.

**Table 3:** Comparative analysis of cold forging simulation vs. experimental results.

Criteria	Simulation Result (Deform 3D)	Experimental Result (Cold Forging Setup)	Validation & Remarks
Preform Geometry	Cylindrical (φ 26 mm × 59 mm) with spherical segment (*r* = 30 mm)	Same geometry used as in simulation	Excellent match; ensures consistency and validity in comparative analysis
Material	Commercially pure Aluminum (99.8%)	Same material used	Validates material behavior assumptions in FEM model
Forging Method	Cold forging under fixed bottom and downward-moving top die	Closed-die cold forging using 100-ton UTM	Real-world process closely mimics simulation parameters
Die Configuration	Hollow spherical cavity with 40 mm diameter	Identical die manufactured using H13 steel	Ensures accurate physical representation of simulation boundary conditions
Frictional Effect	Accounted for via simulation settings (software-defined coefficient)	Evident in ovality of forged product and higher energy demand	Simulation slightly underestimates real-world friction; significant impact on flow and energy
Energy Requirement (*r* = 30 mm)	Lower total energy, linear increase over time (Fig. 8c)	Higher total energy required, especially at start (Fig. 11)	Discrepancy due to strain hardening and real friction; matches literature^16,17^,
Flash Formation	None observed in optimal preform (*r* = 30 mm)	No flash observed	This confirms that a 30 mm spherical radius yields superior results in flash free forging.
Die Filling Quality	Fully filled cavity with some flattening at poles	Good cavity fill, but ovality observed	Both suggest near-complete filling; ovality in experiment due to frictional flow resistance
Surface Finish	Not explicitly stated, assumed ideal due to software visualization	Smooth, defect-free surface observed	Cold forging benefits (no oxidation, minimal defects) confirmed
Shape Accuracy	Near-spherical with flat poles (simulation result, Fig. 3b)	Oval cross-section noted (experiment, Fig. 10)	Validates that friction impacts shape accuracy more in real-world than modeled
Stress Development	Flow stress increases with back stress evident at cavity fill (Fig. 7)	Indirectly confirmed via energy rise and final form resistance	Good alignment in mechanical response under deformation

Table 3 exhibits the good overall agreement that the simulation closely predicts material flow trends, cavity filling and energy consumption profiles. Some discrepancies in energy and final shape are observed; it may be attributed to the friction not perfectly captured in the simulation and strain hardening effects during actual forging. Despite very minor deviations, the experimental results confirm the simulation’s accuracy and reliability, particularly with respect to the refined preform design, process energy efficiency, and flash minimization.

The Table 4 lists different measurable values determined from the user defined parameters used to determine the following, the energy input required for forming a part, the roundness of the final part, whether dies totally filled during forming, whether the volume lost during forming, the path taken by material flow during forming, and the final part dimensions. Examples to support these differences are shown in the Table 3. The forgings required an energy input of 12.8% more than what the FE model had predicted due to both friction and strain hardening, which typically exhibit greater effects in practice.

**Table 4:** Quantitative comparison between simulation and experimental results.

Parameter	Simulation result	Experimental result	Deviation / remarks
Forging Energy	Lower, linear increase (Fig. 7c)	Higher initial and total energy (Fig. 10)	12.8% higher energy in experiment due to friction and strain hardening
Ovality of Final Product	1.9%	3.6%	Difference arises from real frictional resistance in cold forging
Die-Filling Completeness	97.4%	95.1%	Experiment slightly lower due to constraint and surface friction
Flash Formation	None (for *r* = 30 mm preform)	None	Good agreement
Volume Conservation	< 1% error	< 1% error	Indicates accurate FE strain calculations
Shape Accuracy	Near-spherical, slight pole flattening	Ovality visible in top view	Shape deviation linked to real friction and work hardening effects
Material Flow Trend	Correctly predicted (radial constrained)	Matches overall trend	Confirms FE model captures general deformation behaviour

The roundness of the final parts produced from each of the simulated forging processes was comparable visually (within 1.9% from simulated forms compared to experimental roundness of 3.6%). However, due to the presence of friction, the shapes did not maintain a uniform path while flowing through the die cavities. The FE model, even though it did not account for all of the effects of friction, did predict similar deformation patterns, did exhibit a high total die filling ratio (97.4% simulated filling compared to 95.1% actual filling), and volume loss was equivalent to 1% in both cases; therefore, while the FE model does not account for all the frictional and strain hardening effects, it does predict the material deformation patterns effectively and the total flow through the die accurately.

Most of the discrepancies observed between the experimental test and simulation results can be attributed to increased friction and greater amount of strain hardening created during real physical forging, compared to that predicted by the finite element model. Thus, because of the variably poor surface conditions of the material, die/workpiece interaction, and localized deformation present in an actual forge, the resistance to material flow is considerably greater than would be expected with idealistic and simplified boundary conditions^15,16^. Hence, the experiment itself required higher energy to achieve the same result, is slightly more oval than would be expected, and filled the die marginally less than predicted by simulation.

## Conclusions

This work demonstrates the near-net-shape forging of pure aluminium. The important findings are summarized below:

1. Simulation revealed that preform geometry significantly impacts the quality of die filling, flash formation, and final product shape. Cylindrical preforms with spherical ends performed better than rectangular ones, which caused excessive flash and improper cavity filling.
2. The use of DEFORM-3D (V11.2) successfully predicted material flow, deformation patterns, and energy consumption. Simulation results were consistent with experimental outcomes, demonstrating FEA as a reliable tool for preform and die design comparative evaluation.
3. Among tested configurations (*r* = 40 mm, 35 mm, 30 mm), the preform with *r* = 30 mm required the least energy and produced the best near spherical shape without flash. A smaller spherical radius minimized frictional resistance and volume to deform, reducing required forging energy.
4. Ovality observed in the final forged product was resulted from higher friction between die and workpiece and gradual strain hardening during cold forging.
5. Simulations provide a linear energy-time curve, but the actual forging process has proven to use more energy than that predicted by the simulation’s assumptions (simplified friction and hardening). The data obtained from the experiments indicating the enhanced frictional resistance and progressive strain hardening occurring in actual forging.
6. The simulation well matched the material flow, die filling and energy response, with discrepancies in energy (12.8%), ovality (1.9% versus 3.6%) die filling (97.4% versus 95.1%) and volume conservation (< 1%), being primarily due to frictional resistance and strain hardening effects inherent to the physical forging process.
7. The discrepancies between simulation and experiment are mainly due to frictional underestimation and over-simplified initial hardening assumptions. The limitation of the present study is also the absence of calibrated friction-based measurements, and more advanced constitutive modeling should be considered to make better predictions.

### Industrial Relevance, future Research, and application

The validated approach offers a scalable methodology for forging more complex geometries in industrial applications. The methodology promotes cost-effective, energy-efficient, and material-saving practices, alignment with lean manufacturing principles. This investigation lays the groundwork for applying near-net shape forging principles to complex geometries and different material systems. Future work could explore friction management, lubrication strategies, and temperature effects to further refine the process.

## Data Availability

Data is provided within the manuscript.
